# Professional competencies in environmental health in a nursing curriculum from the perspective of students

**DOI:** 10.1590/0034-7167-2024-0502

**Published:** 2025-11-17

**Authors:** Marcela de Abreu Moniz, Lidia Santos Soares, Jane Baptista Quitete, Rosana de Carvalho Castro, Aline Cerqueira Santos Santana da Silva, Ana Claudia Mateus Barreto, Carolina de Alcantara Campos, Núria Suiane dos Santos de Sá

**Affiliations:** I Universidade Federal Fluminense. Rio das Ostras, Rio de Janeiro, Brazil

**Keywords:** Environmental Health, Professional Competence, Nursing, Curriculum, Students, Nursing., Salud Ambiental, Competencia Profesional, Enfermería, Curriculum, Estudiantes de Enfermería

## Abstract

**Objectives:**

to analyze the curricular activities that contributed to the achievement of professional competencies in environmental health in an undergraduate nursing course from the perspective of students.

**Methods:**

a descriptive-exploratory study of a qualitative nature, which used an online form with 22 nursing undergraduates from the last two semesters of the course at a federal university. Data were treated by thematic categorical analysis and descriptive statistics.

**Results:**

two thematic axes emerged: “Fragmented curricular activities and achievement of competencies in environmental health” and “Need for interdisciplinary curricular components in Environmental Health”. The most achieved competency in the students’ perception was the ability to seek collaboration with people and institutions to promote healthy environments (91.3%), and the least achieved was the ability to advocate for environmental justice and reduce health inequities (34.8%).

**Final Considerations:**

curricular changes such as the inclusion of interdisciplinary practices in environmental health were understood by students as indispensable to the training of nurses.

## RESUMEN

**Objetivos:**

analizar las actividades curriculares que contribuyeron al logro de competencias profesionales en salud ambiental en la carrera de Enfermería desde la perspectiva de los estudiantes.

**Métodos:**

estudio descriptivo-exploratorio, de carácter cualitativo, que utilizó un formulario en línea con 22 estudiantes de graduación de enfermería de los dos últimos períodos del curso de una universidad federal. Los datos fueron procesados mediante análisis categórico temático y estadística descriptiva.

**Resultados:**

surgieron dos ejes temáticos: “Actividades curriculares fragmentadas y alcance de las competencias en salud ambiental” y “Necesidad de componentes curriculares interdisciplinarios en Salud Ambiental”. La habilidad más lograda en la percepción de los estudiantes fue la capacidad de buscar colaboración con personas e instituciones para promover entornos saludables (91,3%) y la menos lograda fue abogar por la justicia ambiental y reducir las inequidades en salud (34,8%).

**Consideraciones Finales:**

los cambios curriculares como la inclusión de prácticas interdisciplinarias en salud ambiental fueron entendidos por los estudiantes como esenciales para la formación de enfermeras.

## INTRODUCTION

The COVID-19 pandemic (Coronavirus Disease 2019) was one of the major global health challenges that served as a reminder to humanity, once again, of the intertwined relationship between public health and the environment^(1)^. A triple planetary crisis is threatening the survival not only of humans but of all living beings on the planet in the 21st century. Climate change, extensive biodiversity loss, and pollution in all its forms (air, microplastics, pesticides, heavy metals, etc.) have been identified as some of the greatest health dilemmas on the planet^(2)^. These effects are the result of unsustainable anthropogenic transitions and activities^(1)^. In the Americas, it is estimated that between 8% and 23% of total deaths are attributable to environmental risks/hazards^(3)^.

In this context, it is evident that the global scenario increasingly demands eco-ethical attitudes and good socio-environmental practices from nurses as citizens, leaders, and health professionals engaged in health promotion, the defense of environmental rights and health equity, and mediation in situations of risks, diseases, and health problems resulting from human exposure to environmental imbalances^(4-7)^. Environmental health refers to an important field of public health aimed at analyzing and intervening in the environmental conditions and determinants-physical, chemical, biological, and social-that can influence health, quality of life, and human well-being^(6,8)^.

Nursing care practices for disease prevention and patient health recovery have been intertwined with environmental and personal hygiene measures since the premises of Florence Nightingale’s environmentalist theory over 200 years ago^(9,10)^. However, nurses have not demonstrated sufficient professional skills to implement actions consistent with the challenges and situations encountered in territories shaped by the complex interactions among health, development, and the environment^(4)^. Environmental education has been notably underutilized by nurses in communities, despite being an important work tool that enables the sensitization and involvement of other health professionals and the population regarding the impacts of environmental problems on human health^(11)^.

Recent studies highlight that nurses have struggled to align their daily work processes with the goals set out in the 17 Sustainable Development Goals (SDGs), as per the 2030 Agenda proposed by the United Nations in 2015^(12)^. In this regard, equipping nursing students with knowledge about environmental health, global health, and sustainable development during their undergraduate education is crucial. Such training allows future nurses to develop professional competencies and implement care, managerial, educational, and scientific actions aligned with socio-environmental causes and the improvement of health conditions for populations facing environmental vulnerability^(13,14)^. Nursing professors and researchers worldwide have advocated for nursing education oriented toward environmental health competencies at universities^(14,15)^.

The professional competencies in environmental health required of nurses are outlined by Gilden^(14)^ from the Alliance of Nurses for Healthy Environments (ANHE) and summarized as: the ability to apply environmental health evidence and knowledge; to seek intersectoral partnerships to promote healthy environments; and to develop actions aimed at ensuring environmental rights and health rights for all populations^(14)^. In Brazil, these professional competencies must be tailored to address the environmental, social, and cultural particularities and health needs of population groups marked by vulnerabilities, such as quilombola communities, Indigenous peoples, rural populations, and those living in forests and waterside areas^(16)^.

In this scope, nursing curricula concerning the acquisition of environmental health competencies need to be revised in light of the SDGs, health-disease processes, and planetary health^(10,12)^. However, the international scenario still lacks scientific proposals that expand this debate and investigate aspects of education and curricula that may influence the training of nurses in developing professional competencies in environmental health.

In this context, students’ perceptions serve as a valuable methodological resource in nursing education research, capable of stimulating their participation in evaluating formative processes^(17)^. Such perceptions result from a cognitive process shaped by the knowledge acquired before and during undergraduate studies, as well as an emotional-intuitive process mediated by students’ social, cultural, and psychological experiences^(18)^.

## OBJECTIVES

To analyze the curricular activities that contributed to achieving professional competencies in environmental health within a nursing undergraduate program from the perspective of students.

## METHODS

### Ethical aspects

This study was approved by the Research Ethics Committee for Humanities at *Universidade Federal Fluminense*. Informed Consent was obtained from all individuals involved in the study through an online platform. To maintain participants’ anonymity, alphanumeric identification was used, with the letter E representing “student”, followed by the sequential number of the forms.

### Type of study

This is a descriptive-exploratory study of a qualitative nature, utilizing a semi-structured online self-administered form. The qualitative approach adopts an in-depth and unique analysis of social objects and phenomena based on the meanings, values, perceptions, and experiences of participants, recognizing subjectivity and human plurality^(19)^. Methodological rigor was ensured using the Consolidated Criteria for Reporting Qualitative Research (COREQ) tool^(20)^.

### Methodological procedures

The semi-structured online form included questions regarding social characteristics (gender, age, race, academic period), the achievement of professional competencies in environmental health, participation in curricular activities related to teaching, research, and outreach that contributed to attaining these competencies, and proposals for curriculum changes aimed at improving progress toward these professional competencies.

A closed-ended question (“Mark with an X the response(s) that corresponds to your ability as a future nurse”) was designed to assess the achievement of ten professional competencies in environmental health as defined by Gilden^(14)^: 1. Application of basic environmental health concepts in nursing strategies for assessing, preventing, and controlling environmental health problems; 2. Inclusion of environmental risk factors throughout the human life cycle when assessing the health of individuals, families, and communities; 3. Use of scientific evidence and the principle of environmental precaution; 4. Reduction of environmental health risks in health care settings (chemical, biological, and radiological); 5. Collaboration with other individuals and institutions to create and implement strategies promoting healthy environments; 6. Promotion of healthier environments that respect the diverse values, beliefs, cultures, and circumstances of users, their families, and communities; 7. Advocacy for healthy environments, including issues related to air, water, soil, food/agriculture, the built environment, and chemicals; 8. Promotion of the right to know about the potential harm and risks of products, pollutants, and other hazards to which individuals and communities are exposed; 9. Communication of environmental health risks and promotion of strategies to reduce environmental exposure for individuals, families, and communities; 10. Advocacy for environmental justice, including commitment to the health of vulnerable populations and reducing health inequities.

The use of these analytical dimensions in the construction of the form is justified by the lack of a validated instrument to investigate specific environmental health competencies among nursing students and professionals.

### Study setting

The study was conducted in a nursing bachelor’s degree program at a federal university campus located in a municipality on the coastal lowland region of Rio de Janeiro State. The program was established in 2008 and has a minimum duration of 10 semesters and a maximum of 15 semesters, with a total workload of 4,770 hours^(21)^.

### Data collection and organization

Data collection occurred from March 2022 to April 2023. Invitations were sent to participants via a link to a Google^®^ Forms application containing the Informed Consent Form and the study’s online questionnaire. A database in Excel^®^ spreadsheet format was generated after participants completed the tool.

Participants were approached using their institutional email contacts. Inclusion criteria were: being a student regularly enrolled in one of the following courses: Supervised Internship III (a mandatory course in the ninth period) or Supervised Internship IV (a mandatory course in the tenth period) of the nursing undergraduate program at a federal university campus in the coastal lowland region of Rio de Janeiro State; and being at least 18 years old. The exclusion criterion was: being a nursing student who had withdrawn from the course.

### Data analysis

Data collected through closed-ended questions underwent descriptive statistical analysis, while data from open-ended questions were analyzed qualitatively using Bardin’s categorical content analysis^(22)^.

Content analysis involved sequential steps: pre-analysis (familiarization with the database through reading and rereading, determination of objectives, hypotheses, and indicators), material exploration (coding relevant data and identifying significant themes or registration units), and treatment, inference, and consolidation of the results into categories^(22)^. In the second step, the material’s analysis approach and category definition were deductive, based on prior knowledge. During this coding stage, both explicit and implicit (absent) content were included in the categories, based on semantic (thematic) criteria^(22)^.

These analytical categories emerged into two thematic axes of results: “Fragmented curricular activities and the achievement of environmental health competencies” and “The need for interdisciplinary curricular components in Environmental Health”, based on researchers’ interpretations aligned with the study’s objective^(22)^.

## RESULTS

The study included 22 (100.0%) nursing students aged between 23 and 44 years, with a mean age of 25 years and a predominance of females (82.0%). Specifically, 73.0% of these students were in the ninth semester of the program, while 27.0% were in the tenth semester. Regarding race, 59.0% identified as White, 28.0% as mixed race, and only 13.0% as black.

### Fragmented curricular activities and achievement of competencies in environmental health

The vast majority (91.3%) of the participants reported feeling prepared as future nurses to seek collaboration with others and institutions to create and implement strategies that promote healthy environments, identifying competency number 5 as the most achieved during their training.

The second most frequently reported competency was number 1, with 87.0% of participants indicating their ability to apply basic environmental health knowledge in nursing strategies for risk assessment and prevention of health problems.

This connection between the students and the content of competencies 1 and 5 aligns with the finding that 91.0% of participants mentioned that the mandatory courses in the curriculum contributing to the development of environmental health competencies were related to public/collective health. These courses included Nursing in Collective Health, Nursing in Public and Environmental Health, Policy, Planning, and Health Management, and Public Health Internship. Additionally, 9.1% reported an elective course on Basic Toxicology.

However, participants also highlighted the limited opportunities to apply environmental health concepts in the theoretical-practical teaching of courses and mandatory internships during their training. Some student comments included:

*Incorporating the importance of studying Environmental Education into courses with a practical focus, aimed at raising awareness among the population for a more sustainable society.* (E19)

*We should have more practical activities that integrate knowledge of health and the environment.* (E10)

It was further noted that none of the students in this study achieved all ten professional competencies in environmental health. A contrasting result showed that only a small proportion, 34.8%, reported achieving competency number 10, which involves advocating for environmental justice, including a commitment to the health of vulnerable populations and reducing health inequities, as shown in Chart 1.

**Chart 1:** Ten professional competencies in environmental health achieved during undergraduate studies, according to the perspective of nursing students (N=22), Brazil, 2023

Professional Competencies	n (%)
1. Apply knowledge of basic environmental health concepts to nursing strategies for the prevention and assessment of health risks and problems.	20 (87.0%)
2. Apply knowledge of basic environmental health concepts to nursing strategies for the prevention and assessment of health risks and problems.	18 (78.3%)
3. Use scientific evidence to adopt attitudes and actions guided by the principle of environmental precaution.	15 (65.2%)
4. Reduce chemical, biological, and radiological risks in healthcare settings.	17 (73.9%)
5. Collaborate with others to design and implement strategies that promote healthy environments.	21 (91.3%)
6. Promote healthier environments that respect the diverse values, beliefs, cultures, and circumstances of individuals, their families, and communities in their territories.	17 (73.9%)
7. Advocate for actions that promote healthy environments, addressing issues related to air, water, soil, food/agriculture, built environments, and chemicals.	16 (69.6%)
8. Promote the right to know about the potential harm and risks of products, pollutants, and other hazards to which individuals and communities are exposed.	17 (73.9%)
9. Communicate environmental health risks to individuals, communities, and institutions to develop actions that reduce environmental exposure to hazards (chemical, biological, and physical contaminants).	18 (78.3%)
10. Advocate for environmental justice, including a commitment to the health of vulnerable populations and the reduction of health inequities.	8 (34.8%)

Regarding research, one of the guiding pillars of the nursing curriculum, half (50.0%) of respondents reported participating in research activities that contributed to the development of environmental health competencies during their training. Of these, 22.7% indicated they were members of the university’s Nursing Tutorial Education Program (PET in Portuguese) Group and participated in research and outreach activities related to environmental health.

This highlights that a significant portion of participants in this study do not feel equipped to practice evidence-based nursing in environmental health. This may correlate with the finding that 65.2% reported feeling prepared to use scientific evidence to adopt actions guided by the principle of environmental precaution.

In terms of outreach, 36.4% of students reported participating in outreach activities that contributed to their development of environmental health competencies. These activities included an international symposium on biosafety, a project on truck drivers’ health, a recycling workshop, a lecture on proper disposal of healthcare waste, and activities from the PET Nursing Group.

The students’ comments suggest that curricular activities related to teaching, research, and outreach in environmental health were fragmented and sporadic in the undergraduate program.

### The need for interdisciplinary curricular components in Environmental Health

This category highlights the need for changes in the program’s curricular components, as perceived by the study participants, to develop professional competencies in environmental health during their training.

Some participants expressed a desire to deepen their knowledge and emphasized the need to incorporate environmental health content into mandatory courses or even create a specific course for this purpose, as reflected in the following comments:

*I believe expanding environmental health content within the curriculum would provide us with a broader knowledge base on environmental health.* (E7)

*Creating a mandatory course that addresses environmental health.* (E15)

From the perspective of a multifaceted and interdisciplinary approach to environmental health, some students suggested incorporating topics through theoretical and practical teaching in courses not exclusively related to collective health nursing, as seen in the following statements:

*The Occupational Health course could contribute to this topic, integrating theory and practice with the risks of irresponsible disposal of medications, injections, and materials in inappropriate places.* (E14)

*Environmental health could be addressed in courses other than Public Health, integrating it into other topics.* (E5)

*More practical activities in courses that integrate knowledge of health and the environment.* (E11)

*There could be courses on health and the environment.* (E4)

Complementary outreach and teaching activities should be offered as strategic actions for nursing education to enhance students’ knowledge and skills in environmental health critically, as suggested by one participant:

*Promoting training courses aimed at encouraging a critical view of environmental health for students.* (E11)

## DISCUSSION

The segmented approach to environmental health in mandatory courses and curricular activities during undergraduate nursing programs appears to have failed to equip students with all the professional competencies in environmental health explored in this study.

The National Curricular Guidelines for undergraduate nursing programs in Brazil highlight environmental health as an essential field to be addressed through an integrated approach between theory and practice, emphasizing that topics related to environmental education should be transversal within the nursing curriculum^(23,24)^. These guidelines stipulate that by the end of their education, professionals should demonstrate general and specific competencies to perform nursing work processes consistent with contemporary socio-environmental demands^(23)^. Despite the legal framework’s emphasis on addressing environmental issues in nursing higher education in Brazil, the field presents weaknesses regarding the development of competencies aimed at an environmentalist, ethical, and holistic approach to care at the microdimension-local, school, university, workplace, home, city-and macrodimension-country, continent, planet^(23-27)^.

In this study, nursing students reported that environmental health has been applied sporadically and is limited to public health-related courses during their undergraduate education. This concern aligns with studies showing that environmental aspects are fragmented and taught from a technicist and linear perspective within the curriculum, failing to promote meaningful changes in awareness, critical thinking, or interest in the subject^(24,28)^.

Moreover, the students in this study highlighted the need for curricular transformation based on interdisciplinarity. This perspective aligns with authors who argue that the content of courses addressing environmental health should be interconnected and complementary, fostering a more global and in-depth understanding of topics in this field^(13,24,28)^.

Environmental issues have been incorporated into specific courses within nursing curricula without standardization in their delivery or integration of content and activities across courses in different academic terms^(24)^. This curricular approach reveals challenges in overcoming the disciplinary focus of nursing education processes due to limited societal understanding of the intersections between health, environment, and nursing^(6)^.

In this context, it is essential to prioritize a research and teaching agenda in nursing that incorporates new topics transversally, such as the SDGs^(26)^, global health competencies^(15)^, and educational strategies that stimulate critical thinking about the relationship between health and the environment. This would help transcend the care-centered, curative model in training environments, benefiting undergraduate and postgraduate nursing students and nurse educators alike^(13,24,26-29)^.

The potential of transdisciplinarity in environmental health education stands out, fostering communication and collaboration among different fields of knowledge in nursing education^(29)^. Addressing educational gaps is feasible through interdisciplinary and transversal environmental education activities using innovative and nontraditional methodologies that aim to encompass the singularities of local and global environmental health^(17)^.

One participant in this study emphasized the need for a critical perspective on environmental health education in the context of nursing training. This perspective is consistent with authors who highlight that achieving competency in environmental health is promising through the robust incorporation of activities promoting critical, emancipatory, and transformative environmental education into nursing curricula^(4,17)^.

In light of this, it is imperative to critically examine aspects of environmental education, such as eco-literacy and an ecosystemic approach to health, employing active teaching methodologies with a critical approach during undergraduate studies to prepare future nurses to transform processes in popular and continuing health education^(6,8,10)^.

It is also important to reflect on the findings of this study, which indicate that students were only provided with an introductory or elementary view of environmental health during their training, particularly regarding participation in situations of environmental health injustice. Nurses need to seek advanced knowledge about environmental hazards and their health effects and become capable of advocating for environmental justice in communities^(30)^.

To this end, these professionals must understand and collaborate on addressing environmental issues through political actions related to health and sustainability^(31)^. Therefore, nursing educators must prioritize curricular reforms that prepare future professionals with leadership, governance, and health management skills aligned with the goals of promoting health equity and environmental equity across all populations on our planet^(28,31)^.

According to the Pan American Health Organization^(32)^, one of the strategic directives for nursing in the Americas is to strengthen the quality of nursing education to develop nurse leaders capable of collaborating with the SDGs and enabling efficient health systems and service responses to local and global health disparities.

Environmental injustices among social groups are exacerbated by unequal access to healthcare services, sanitation, leisure, and well-being resources, which are essential for preventing and effectively responding to diseases, crises, and health emergencies^(2)^. In this context, transcultural nursing has advanced studies and interventions in health and the environment to implement and evaluate policies and practices aimed at reducing health inequities among groups experiencing greater socio-environmental vulnerability^(33)^.

For instance, the climate emergency is a pressing situation that places some populations at a disadvantage, particularly regarding the capacity of health services to address the various health issues caused by climate-related disasters^(6,34)^. Some educational initiatives involve collaborative actions among non-governmental organizations, universities, and schools, showing great potential to foster engagement and empower children and young people on various aspects of resilience and climate justice^(34)^.

Nurses have an ethical responsibility to promote global health and should, therefore, intervene in mitigating climate risks that negatively impact individual and collective health, as stated by the International Council of Nurses^(9)^. Within this scope, nurses play a crucial role in creating opportunities and seeking intersectoral partnerships to develop emancipatory practices in environmental health^(4)^, thereby reinforcing an ethical approach to environmental responsibility and health rights^(7,9)^.

From a systemic, pluralistic, and bioethical perspective, nursing competencies should support the implementation of best practices in assessing, monitoring, and reducing environmental health risks, such as nursing processes, participatory socio-environmental diagnoses, and citizen education, considering territorial and global health issues^(4,7,10)^.

It is necessary for various associations and educational and health institutions to provide activities and events that stimulate debate, raise awareness, and promote critical thinking among nursing students and professionals about issues related to environmental health and sustainable development. This aligns with the proposals of the ANHE^(14)^, ANHE Latin America^(9)^, and the Brazilian Nursing Association in 2023^(35)^. Examples like sustainable universities and health-promoting universities also contribute to fostering ethical environmental health mindsets and attitudes in university settings^(36,37)^.

Therefore, there is an urgent need to promote more spaces in universities that provide effective support for the emancipatory training of nurses as critical and strategic agents of change, capable of innovating, developing, adapting, and envisioning new studies, models, policies, and practices of care that are committed to health, environmental ethics, and the sustainability of life in the face of the emerging challenges posed by environmental impacts on local and global health.

### Study limitations

One limitation pertains to the study being conducted with a single group of undergraduate nursing students from one higher education institution. Another limitation relates to the use of a qualitative methodological approach and the results being based on students’ subjective perceptions, which were detached from an objective assessment of the professional competencies they reported achieving.

### Contributions to the field of Nursing

This study highlights the need for curricular reform aimed at more effectively integrating concepts and practices of environmental health care into nursing education. Furthermore, it underscores the importance of raising awareness among nursing undergraduates about contemporary and complex environmental health issues, such as environmental justice and health equity, to foster more conscientious, innovative, and eco-ethical practices among future health professionals that can impact both local and global settings.

Thus, this research aligns with the commitment to strengthening the education and professional practices of nursing in environmental health and has the potential to contribute to enhancing the interdisciplinary approach to environmental health in curricula, emphasizing the value of nursing science, education, and profession in the context of global health, public health, and environment in Brazil and the Americas.

## FINAL CONSIDERATIONS

Curricular changes, such as the inclusion of interdisciplinary teaching practices in environmental health, were deemed indispensable by students for nurse training. The reflections in this study on the potential of adopting curricular strategies in environmental health in nurse training contexts support the pathway toward breaking away from educational practices based solely on informational and instrumental actions and methodologies. These changes aim to inspire a critical perspective and solidarity among future nurses in the pursuit of more just, sustainable, and healthy societies.

## Data Availability

The research data are available within the article.
