# Supplementary material for: Professional competencies in environmental health in a nursing curriculum from the perspective of students
Source: Rev Bras Enferm. 2025 Nov 17;78(Suppl 3):e20240502. doi: 10.1590/0034-7167-2024-0502 (PMC12622972; doi:10.1590/0034-7167-2024-0502)
Supplement: Translation

# Translation: Competências profissionais em saúde ambiental em um currículo de Enfermagem na perspectiva de estudantes

**DOI:** 10.1590/0034-7167-2024-0502pt

**Authors:** Marcela de Abreu Moniz, Lidia Santos Soares, Jane Baptista Quitete, Rosana de Carvalho Castro, Aline Cerqueira Santos Santana da Silva, Ana Claudia Mateus Barreto, Carolina de Alcantara Campos, Núria Suiane dos Santos de Sá

**Affiliations:** I Universidade Federal Fluminense. Rio das Ostras, Rio de Janeiro, Brasil

**Keywords:** Saúde Ambiental, Competência Profissional, Enfermagem, Currículo, Estudantes de Enfermagem.

## Abstract

**Objetivos:**

analisar as atividades curriculares que contribuíram para o alcance de competências profissionais em saúde ambiental em um curso de graduação em Enfermagem, na perspectiva de estudantes.

**Métodos:**

estudo descritivo-exploratório, de natureza qualitativa, que empregou formulário on-line com 22 graduandos de Enfermagem dos dois últimos períodos do curso de uma universidade federal. Os dados foram tratados por meio de análise categorial temática e estatística descritiva.

**Resultados:**

emergiram dois eixos temáticos: “Atividades curriculares fragmentadas e alcance de competências em saúde ambiental” e “Necessidade de componentes curriculares interdisciplinares em Saúde Ambiental”. A competência mais alcançada, na percepção dos estudantes, foi a capacidade de buscar colaboração com pessoas e instituições para promover ambientes saudáveis (91,3%); e a menos alcançada foi a de advogar pela justiça ambiental e redução das iniquidades em saúde (34,8%).

**Considerações Finais:**

mudanças curriculares, como a inclusão de práticas interdisciplinares em saúde ambiental, foram compreendidas pelos estudantes como indispensáveis à formação dos enfermeiros.

## INTRODUÇÃO

A pandemia de COVID-19 (*Coronavirus Disease 2019*) foi um dos grandes problemas de saúde planetária que serviu para alertar a humanidade, novamente, sobre a relação imbricada entre a saúde pública e o meio ambiente^(1)^. Uma tripla crise planetária vem ameaçando a sobrevivência não apenas dos seres humanos, mas de todos os seres vivos planetários no século XXI. As mudanças climáticas, a extensa perda de biodiversidade e a poluição em todas as suas formas (ar, microplásticos, agrotóxicos, metais pesados etc.) têm sido apontadas como alguns dos maiores dilemas de saúde do planeta^(2)^. Tais efeitos são resultados das transições e atividades antropogênicas insustentáveis^(1)^. Na Região das Américas, avalia-se que entre 8% e 23% do total de mortes sejam atribuídas aos riscos/perigos ambientais^(3)^.

Nesse sentido, depreende-se que o cenário global vem exigindo cada vez mais atitudes ecoéticas e boas práticas socioambientais pelos enfermeiros enquanto cidadãos, líderes e profissionais da saúde engajados com a promoção da saúde, a defesa dos direitos ambientais e da equidade em saúde, e a mediação em situações de riscos, doenças e agravos à saúde decorrentes da exposição humana aos fatores de desequilíbrio ambiental^(4-7)^. A saúde ambiental refere-se a um campo importante da saúde pública que tem por finalidade a análise e a intervenção das condições e determinantes ambientais nas dimensões física, química, biológica e social, que podem exercer influência sobre a saúde, a qualidade de vida e o bem-estar humano^(6,8)^.

As práticas cuidativas para prevenção de doenças e recuperação da saúde dos pacientes pela Enfermagem estão interligadas às medidas de higiene ambiental e individual desde os pressupostos da teoria ambientalista de Florence Nightingale, há mais de 200 anos^(9,10)^. Todavia, os enfermeiros não têm demonstrado habilidades profissionais para a realização de ações condizentes com os desafios e as situações encontradas nos territórios produzidas pelas complexas interações entre saúde, desenvolvimento e ambiente^(4)^. A educação ambiental, notadamente, tem sido pouco utilizada pelos enfermeiros nas comunidades, apesar de se apresentar como uma ferramenta de trabalho importante, que possibilita sensibilizar e envolver outros profissionais da saúde e a população sobre os impactos dos problemas ambientais na saúde humana^(11)^.

Estudos hodiernos evidenciam que enfermeiros não têm conseguido vincular seus processos de trabalho cotidianos com as metas estabelecidas nos 17 Objetivos do Desenvolvimento Sustentável (ODS), conforme a Agenda 2030, proposta pela Organização das Nações Unidas em 2015^(12)^. Nesta linha de pensamento, a instrumentalização de estudantes sobre saúde ambiental, saúde global e desenvolvimento sustentável no decorrer do curso de graduação em Enfermagem é fundamental para que estes futuros enfermeiros desenvolvam competências profissionais e, assim, consigam implementar ações assistenciais, gerenciais, educativas e científicas envolvidas com a causa socioambiental e a melhoria das condições de saúde de populações em situação de vulnerabilidade ambiental^(13,14)^. Professores e pesquisadores de Enfermagem de diversas partes do mundo têm motivado a educação em Enfermagem orientada por competências em saúde ambiental nas universidades^(14,15)^.

As competências profissionais em saúde ambiental necessárias ao enfermeiro são sinalizadas por Gilden^(14)^, da Aliança de Enfermeiros por Ambientes Saudáveis (*Alliance of Nurses for Healthy Environments - ANHE*), e sintetizadas como: saber aplicar evidências e conhecimentos de saúde ambiental; buscar parcerias intersetoriais para promover ambientes saudáveis; e desenvolver ações com a finalidade de garantir direitos ambientais e direitos à saúde para todas as populações^(14)^. No território brasileiro, concebe-se que tais competências profissionais devem estar ajustadas para atender às particularidades ambientais, sociais e culturais e às necessidades de saúde de grupos populacionais marcados por vulnerabilidades, tais como quilombolas, indígenas, populações do campo, da floresta e das águas^(16)^.

Neste escopo, os currículos de Enfermagem, em relação à obtenção de competências em saúde ambiental, precisam ser revisados frente aos ODS, aos processos saúde-doença e à saúde planetária^(10,12)^. Contudo, o cenário internacional ainda carece de propostas científicas que ampliem esse debate e investiguem aspectos do ensino e do currículo que podem interferir na formação dos enfermeiros no que tange ao desenvolvimento de competências profissionais em saúde ambiental.

Nesse contexto, a percepção de estudantes é um dos recursos metodológicos valiosos de pesquisa em educação em Enfermagem, que pode estimular a participação desses indivíduos na avaliação de processos formativos^(17)^. Tal percepção é resultante de processo cognitivo originado pelo processamento de conhecimentos adquiridos antes e durante a graduação, e emocional-intuitivo, mediado por experiências sociais, culturais e psicológicas do estudante^(18)^.

## OBJETIVOS

Analisar as atividades curriculares que contribuíram para o alcance de competências profissionais em saúde ambiental em um curso de graduação em Enfermagem, na perspectiva de estudantes.

## MÉTODOS

### Aspectos éticos

O presente estudo foi aprovado pelo Comitê de Ética em Pesquisa da área de Humanas da Universidade Federal Fluminense. O Consentimento Livre e Esclarecido foi obtido de todos os indivíduos envolvidos no estudo pela modalidade *on-line*. Para manter o anonimato dos participantes, fez-se a identificação alfanumérica com a letra E, de estudante, seguida do numeral de sequência dos formulários.

### Tipo de estudo

Trata-se de um estudo descritivo-exploratório, de natureza qualitativa, com o emprego de formulário *on-line* semiestruturado por autopreenchimento. A abordagem qualitativa adota uma análise profunda e singular dos objetos e fenômenos sociais, a partir de significados, valores, percepções e vivências dos participantes, reconhecendo a subjetividade e a pluralidade humana^(19)^. O rigor metodológico foi conduzido pela ferramenta *Consolidated Criteria for Reporting Qualitative Research* (*COREQ*)^(20)^.

### Procedimentos metodológicos

O formulário *on-line* semiestruturado contemplou questões referentes às características sociais (sexo, idade, raça, período do curso de graduação), ao alcance das competências profissionais em saúde ambiental, à participação em atividades curriculares de ensino, pesquisa e extensão que contribuíram para o alcance dessas competências e às propostas de mudanças no currículo do curso de graduação, com vistas à progressão para tais competências profissionais.

Uma questão fechada (“Assinale com X a(s) resposta(s) que corresponde(m) à sua capacidade como futuro(a) enfermeiro(a)”) foi estabelecida para identificação do alcance das dez competências profissionais em saúde ambiental, que foram estabelecidas por Gilden^(14)^, a seguir:1. Aplicação do conhecimento de conceitos básicos de saúde ambiental em estratégias de avaliação, prevenção e controle de problemas de saúde ambiental pela Enfermagem; 2. Inclusão de fatores de risco ambientais em todo o ciclo de vida humana ao avaliar a saúde de indivíduos, famílias e comunidades; 3. Uso de evidências científicas e do princípio da precaução ambiental; 4. Redução de riscos de saúde ambiental nos contextos de cuidado em saúde (químicos, biológicos e radiológicos); 5. Busca de colaboração com outras pessoas e instituições para criar e implementar estratégias que promovam ambientes saudáveis; 6. Promoção de ambiente mais saudável que respeite os diversos valores, crenças, culturas e circunstâncias de usuários, suas famílias e comunidades; 7. Defesa de ambientes saudáveis que incluam questões associadas ao ar, água, solo, alimentos/agricultura, ambiente construído e produtos químicos; 8. Promoção do direito de saber sobre o potencial nocivo e os riscos de produtos, poluentes e outros perigos aos quais indivíduos e comunidades estão expostos; 9. Comunicação de riscos em saúde ambiental e promoção de estratégias de redução da exposição ambiental com as pessoas, famílias e comunidades; 10. Defesa da justiça ambiental, incluindo o compromisso com a saúde das populações vulneráveis e a redução das iniquidades em saúde.

É importante justificar o uso dessas dimensões analíticas na construção do formulário, mediante a inexistência de um instrumento validado para investigação sobre competências específicas em saúde ambiental entre estudantes e profissionais de Enfermagem.

### Cenário do estudo

O cenário é um curso de graduação para formação em bacharelado em Enfermagem de uma universidade federal, em um campus localizado em um município da Baixada Litorânea do estado do Rio de Janeiro. Tal curso foi criado em 2008, possui duração mínima de 10 e máxima de 15 semestres letivos para integralização, com uma carga horária total de 4.770 horas^(21)^.

### Coleta e organização dos dados

A etapa de coleta de dados ocorreu no período de março de 2022 a abril de 2023. Os convites foram enviados aos participantes com um link que direcionava para o aplicativo *Google^®^ Forms*, com acesso ao Termo de Consentimento Livre e Esclarecido e ao formulário *on-line* da pesquisa. Um banco de dados em formato de planilha do *Excel^®^* foi gerado após o autopreenchimento dessa ferramenta pelos participantes do estudo.

Os participantes foram abordados utilizando seus contatos institucionais por e-mail. Os critérios de inclusão foram: ser estudante regularmente matriculado em uma das seguintes disciplinas: Estágio Supervisionado III (disciplina obrigatória do nono período) ou Estágio Supervisionado IV (disciplina obrigatória do décimo período) do curso de graduação em Enfermagem de uma universidade federal com campus localizado em um município da região da Baixada Litorânea do estado do Rio de Janeiro; e ter idade a partir dos dezoito anos. Adotou-se como critério de exclusão: ser acadêmico de Enfermagem que realizou trancamento da disciplina.

### Análise dos dados

Os dados coletados mediante as perguntas fechadas receberam tratamento quantitativo de estatística descritiva, e os dados oriundos das perguntas abertas receberam tratamento qualitativo, à luz da análise categorial de conteúdo de Bardin^(22)^.

A análise de conteúdo dos dados foi realizada envolvendo as etapas sequenciais de pré-análise (familiarização com o banco de dados a partir da leitura e releitura, determinação de objetivos, hipóteses e indicadores), exploração do material (codificação dos dados relevantes e surgimento de temas significativos - unidades de registro) e, por fim, tratamento, inferência e consolidação das categorias dos resultados^(22)^. Cabe ressaltar que, na segunda etapa, a abordagem de análise dos materiais e a definição das categorias foram dedutivas, em função de conhecimentos prévios. Nessa etapa de codificação, foram considerados conteúdos explícitos e conteúdos ocultos (ausentes) para inclusão nas categorias, baseadas em critério semântico (temático)^(22)^.

Tais categorias analíticas emergiram em dois eixos temáticos dos resultados: “Atividades curriculares fragmentadas e alcance de competências em saúde ambiental” e “Necessidade de componentes curriculares interdisciplinares em Saúde Ambiental”, por meio de interpretações dos pesquisadores e baseadas no objetivo do estudo^(22)^.

## RESULTADOS

Participaram do estudo 22 (100,0%) estudantes de Enfermagem, com faixa etária de 23 a 44 anos, média de 25 anos, e predominância do sexo feminino (82,0%). Especificamente, 73,0% desses alunos estavam no nono período do curso, enquanto 27,0% estavam no décimo período. Em termos de raça, 59,0% se autodeclararam brancos, 28,0% como pardos, e apenas 13,0% como negros.

### Atividades curriculares fragmentadas e alcance de competências em saúde ambiental

A grande maioria (91,3%) dos participantes deste estudo afirmou que se sente preparada, enquanto futuros enfermeiros, para buscar colaboração com outras pessoas e instituições para criar e implementar estratégias que promovam ambientes saudáveis, revelando a competência de número 5 como a mais alcançada durante o processo formativo.

A segunda competência com maior frequência de respostas foi a de número 1, com 87,0%, que se refere à capacidade de aplicar conhecimentos básicos de saúde ambiental em estratégias de prevenção e avaliação de riscos e problemas de saúde pela Enfermagem.

Nota-se que a aproximação dos estudantes com conteúdos relativos às competências 1 e 5 tem relação com o resultado de que 91,0% dos participantes deste estudo mencionaram que as disciplinas obrigatórias da matriz curricular do curso, que contribuíram para o desenvolvimento de competências em saúde ambiental, estavam relacionadas à saúde coletiva/saúde pública. Dentre essas disciplinas, destacam-se: Enfermagem em Saúde Coletiva; Enfermagem em Saúde Pública e Meio Ambiente; Política, Planejamento e Gestão em Saúde; Estágio Curricular em Saúde Pública. Além disso, 9,1% mencionaram uma disciplina optativa: Noções Básicas de Toxicologia.

Todavia, os participantes também relataram poucas oportunidades de aplicabilidade da saúde ambiental no ensino teórico-prático de disciplinas e estágios obrigatórios ao longo do percurso formativo. Seguem alguns relatos dos estudantes:

*Incorporar a importância de estudar Educação Ambiental nas disciplinas que abrangem o campo prático, com foco em conscientizar a população em busca de uma sociedade mais sustentável.* (E19)

*Deveríamos ter mais atividades práticas que proporcionem assimilar o conhecimento entre saúde e meio ambiente.* (E10)

Percebeu-se, ainda, que não foi atingido o alcance das dez competências profissionais em saúde ambiental por nenhum estudante do presente estudo. Outro resultado contrastante foi que apenas uma pequena parcela dos estudantes, 34,8%, respondeu sobre a competência número 10, que corresponde à capacidade de advogar pela justiça ambiental, incluindo ter compromisso com a saúde das populações vulneráveis e com a redução das iniquidades em saúde, conforme se observa no Quadro 1.

**Quadro 1:** Dez competências profissionais em saúde ambiental alcançadas durante a graduação, segundo a perspectiva de estudantes de Enfermagem (N = 22), Brasil, 2023

Competências profissionais	n (%)
1. Aplicar conhecimentos sobre conceitos básicos de saúde ambiental em estratégias de ação de prevenção e avaliação de riscos e problemas de saúde pela enfermagem	20 (87,0%)
2. Incorporar fatores de risco ambientais em todo o ciclo de vida ao avaliar a saúde de indivíduos, famílias e comunidades	18 (78,3%)
3. Utilizar evidências científicas para adotar atitudes e ações norteadas pelo princípio da precaução ambiental	15 (65,2%)
4. Reduzir riscos químicos, biológicos e radiológicos nos contextos de cuidado em saúde	17 (73,9%)
5. Colaborar com outras pessoas para criar e implementar estratégias que promovam ambientes saudáveis	21 (91,3%)
6. Promover ambientes mais saudáveis condizentes com o respeito dos diversos valores, crenças, culturas e circunstâncias dos indivíduos, suas famílias e comunidades nos territórios	17 (73,9%)
7. Defender ações de promoção de ambientes saudáveis que incluam questões associadas ao ar, água, solo, alimentos / agricultura, ambiente construído e produtos químicos	16 (69,6%)
8. Promover o direito de saber sobre o potencial nocivo e os riscos de produtos, poluentes e outros perigos que os indivíduos e as comunidades estão expostas	17 (73,9%)
9. Comunicar riscos em saúde ambiental às pessoas, comunidades e instituições envolvidas de modo a desenvolver ações para redução da exposição ambiental aos perigos (contaminantes químicos, biológicos e físicos)	18 (78,3%)
10. Advogar pela justiça ambiental, incluindo ter compromisso com a saúde das populações vulneráveis e a redução das iniquidades em saúde	8 (34,8%)

No que se refere à pesquisa, um dos eixos norteadores do currículo da Enfermagem, metade (50,0%) dos respondentes relataram que participaram de alguma atividade de pesquisa que contribuiu para o desenvolvimento de competências em saúde ambiental durante seu processo formativo. Desses participantes, 22,7% revelaram que foram alunos integrantes de um Grupo do Programa de Educação Tutorial (PET) Enfermagem da universidade e participaram de atividades de pesquisa e extensão consoantes com a saúde ambiental.

Desse modo, revela-se que uma parcela importante dos participantes deste estudo não se sente capacitada para realizar práticas de Enfermagem em saúde ambiental baseadas em evidências científicas. Tais resultados podem estar concatenados com o fato de que 65,2% responderam que estão preparados para utilizar evidências científicas a fim de adotar atitudes e ações norteadas pelo princípio da precaução ambiental.

Em relação à extensão curricular, 36,4% dos estudantes deste estudo mencionaram que participaram de alguma ação extensionista que contribuiu para a formação de competências em saúde ambiental, e descreveram as seguintes atividades: simpósio internacional de biossegurança, projeto sobre saúde dos caminhoneiros, oficina sobre reciclagem, palestra sobre descarte correto de resíduos de serviços de saúde, atividades do grupo PET Enfermagem.

Destarte, os relatos dos estudantes deste estudo indicam que as atividades curriculares vinculadas ao ensino, à pesquisa e à extensão em saúde ambiental apresentaram-se fragmentadas e pontuais no curso de graduação em tela.

### Necessidade de componentes curriculares interdisciplinares em Saúde Ambiental

Na presente categoria de resultado, são apontadas as necessidades de mudanças em componentes curriculares do curso, conforme a percepção dos estudantes do presente estudo, com vistas ao desenvolvimento de competências profissionais em saúde ambiental no contexto formativo.

Nesse caminho, verificou-se que foi suscitado, por alguns participantes, o desejo de aprofundamento dos conhecimentos, evidenciando a necessidade de incorporação de conteúdos de saúde ambiental às disciplinas obrigatórias do curso e até mesmo da criação de uma disciplina específica para essa finalidade, conforme os discursos a seguir:

*Acredito que a ampliação de conteúdos voltados à saúde ambiental dentro da grade curricular proporcionaria com que tivéssemos uma maior bagagem de conhecimentos acerca da saúde ambiental.* (E7)

*Criar uma disciplina obrigatória que aborda saúde ambiental.* (E15)

Na perspectiva da abordagem multiforme e interdisciplinar em saúde ambiental, a inclusão de temas, por meio do ensino teórico e prático, em disciplinas da matriz curricular do curso que não sejam exclusivamente da área de Enfermagem em Saúde Coletiva foi percebida por alguns graduandos como uma questão que precisa ser considerada durante a formação do enfermeiro, de acordo com algumas falas:

*A disciplina de saúde do trabalhador poderia contribuir com a temática. Entrelaçando a teoria e prática com os riscos de uma prática irresponsável de descarte de medicamentos, injeções e materiais em lugares inadequados.* (E14)

*A saúde ambiental poderia ser abordada em outras disciplinas além da Saúde Pública, assim incorporando-a em outras temáticas.* (E5)

*Mais atividades práticas em disciplinas que proporcionem assimilar o conhecimento entre saúde e meio ambiente.* (E11)

*Podia ter disciplinas sobre saúde e meio ambiente.* (E4)

Atividades extensionistas e de ensino complementares devem ser ofertadas como ações estratégicas de educação em enfermagem para a ampliação dos conhecimentos e habilidades dos alunos em saúde ambiental de modo crítico, conforme sugere um participante:

*Promoção de cursos de capacitação visando estimular um olhar crítico em saúde ambiental para os alunos.* (E11)

## DISCUSSÃO

A abordagem ambiental de forma segmentada em disciplinas obrigatórias e atividades curriculares durante o curso de graduação em Enfermagem parece não ter proporcionado aos estudantes o alcance de todas as competências profissionais em saúde ambiental exploradas no presente estudo.

As Diretrizes Curriculares Nacionais do curso de graduação em Enfermagem expressam a saúde ambiental como um campo essencial a ser trabalhado de forma articulada entre teoria e prática, e que os temas relativos à educação ambiental devem ser transversais no currículo da Enfermagem^(23,24)^. Tais diretrizes determinam que, ao final da formação, o profissional apresente competências gerais e específicas para desempenhar processos de trabalho em Enfermagem coerentes com as demandas socioambientais contemporâneas^(23)^. Apesar dos indicativos no arcabouço legal sobre a abordagem ambiental no ensino superior de Enfermagem no Brasil, este apresenta fragilidades relativas à formação de competências direcionadas a um enfoque ambientalista, ético e integral de cuidado na microdimensão: local, escola, universidade, trabalho, casa, cidade; e macrodimensão: país, continente, planeta^(23-27)^.

No presente estudo, os graduandos referiram que a saúde ambiental vem sendo aplicada de forma pontual e restrita às disciplinas de saúde coletiva durante o curso de graduação. Tal preocupação dos participantes é corroborada por estudos que demonstram que os aspectos ambientais têm sido instrumentalizados em disciplinas de modo fragmentado e baseados em um enfoque tecnicista e linear no currículo, sem despertar mudanças positivas de sensibilização, criticidade ou interesse sobre o tema^(24,28)^.

Evidenciou-se, ainda, que os estudantes deste estudo sinalizaram para a necessidade de transformação curricular baseada na interdisciplinaridade. Tal pensamento coaduna com autores que reconhecem que os conteúdos das disciplinas acerca da saúde ambiental deveriam se conectar e se complementar, para que o ensino pudesse fluir para uma compreensão mais global e aprofundada sobre os temas desse campo^(13,24,28)^.

As questões ambientais têm sido ofertadas em disciplinas específicas nos currículos de Enfermagem, não existindo uma padronização dessa oferta e tampouco uma articulação dos conteúdos e atividades entre essas disciplinas em diferentes períodos letivos^(24)^. Esse direcionamento curricular desvela impasses para se superar o enfoque disciplinar dos processos educativos da Enfermagem, em razão do próprio debate lacônico e da compreensão limitada da sociedade sobre a saúde, o ambiente e a Enfermagem^(6)^.

Nesse sentido, deve-se abarcar uma agenda de prioridades em pesquisa e ensino de Enfermagem, com a incorporação, de modo transversal, de novos temas, tais como os ODS^(26)^ e as competências em saúde global^(15)^, e de estratégias educacionais que estimulem o pensamento crítico a respeito da relação entre saúde e meio ambiente, superando a lógica do modelo assistencialista-curativista nos espaços formativos, tanto entre graduandos e pós-graduandos em Enfermagem, quanto entre docentes enfermeiros^(13,24,26-29)^.

Destaca-se a potência da transdisciplinaridade na educação em saúde ambiental, fomentando a comunicação e a cooperação entre diferentes áreas do conhecimento na formação do enfermeiro^(29)^. É factível, no preenchimento das lacunas educacionais, a realização de atividades interdisciplinares e transversais de educação ambiental com metodologias inovadoras e não tradicionais, que procurem abranger as singularidades da saúde ambiental local e planetária^(17)^.

Um participante deste estudo ressaltou a necessidade de um olhar crítico voltado à educação em saúde ambiental no contexto formativo do enfermeiro. Tal entendimento é condizente com apontamentos de autores que salientam que a consecução da competência em saúde ambiental é promissora a partir da incorporação, de forma robusta, nos currículos de Enfermagem, de atividades que abarquem a educação ambiental crítica, emancipadora e transformadora^(4,17)^.

À vista disso, é impreterível a problematização de vertentes da educação ambiental, tais como a ecoalfabetização e a abordagem ecossistêmica em saúde, com aplicação de metodologias ativas de ensino de abordagem crítica durante a graduação, com vistas a capacitar o futuro enfermeiro a transformar processos de educação popular e educação permanente em saúde^(6,8,10)^.

Importante refletir também sobre os resultados deste estudo ao indicarem que apenas uma visão introdutória ou elementar da saúde ambiental foi proporcionada no percurso formativo, sobretudo no tocante à participação em situações de injustiça em saúde ambiental. Enfermeiros precisam buscar elevar seus conhecimentos sobre as questões que permeiam os perigos ambientais e seus efeitos à saúde, e se tornarem capazes de interceder pela justiça ambiental nas comunidades^(30)^.

Para tanto, esses profissionais precisam compreender e colaborar no manejo das questões ambientais com ações políticas de saúde e sustentabilidade^(31)^. Portanto, educadores em Enfermagem precisam priorizar reformas curriculares que visem ao preparo dos futuros profissionais na capacidade de liderança, governança e gestão da saúde, alinhada com os propósitos de promoção da equidade em saúde e equidade ambiental em todas as populações de nosso planeta^(28,31)^.

Segundo a Organização Pan-Americana da Saúde^(32)^, uma das diretrizes estratégicas para a Enfermagem na Região das Américas consiste em consolidar a qualidade da educação em Enfermagem para formar enfermeiros líderes, de modo que colaborem com os ODS e com respostas eficientes dos sistemas e serviços de saúde frente às disparidades em saúde locais e globais.

As injustiças ambientais entre os grupos sociais são amplificadas pelo acesso desigual aos serviços de saúde, saneamento, lazer e bem-estar, dificultando a prevenção e a resposta efetiva a doenças, crises e emergências de saúde^(2)^. Com base nessa lógica, a Enfermagem transcultural vem avançando na direção de estudos e intervenções em saúde e ambiente, para implementação e avaliação de políticas e práticas de modo a reduzir as iniquidades em saúde de grupos em situação de maior vulnerabilidade socioambiental^(33)^.

A título de exemplo, a emergência climática é uma das situações prementes que colocam algumas populações em desvantagem, especialmente no que concerne à capacidade dos serviços de saúde em atender os diversos problemas de saúde gerados pelos desastres climáticos^(6,34)^. Algumas iniciativas educativas referem-se à ação colaborativa entre organizações não governamentais, universidades e escolas, e apresentam grande potencial de favorecer o engajamento e o empoderamento de crianças e jovens sobre diferentes aspectos de resiliência e justiça climática^(34)^.

Enfermeiros possuem a responsabilidade ética de promover a saúde global e, assim, devem intervir na mitigação de riscos climáticos que causem efeitos negativos na saúde individual e coletiva, conforme declara o Conselho Internacional de Enfermeiros^(9)^. Neste ensejo, o enfermeiro tem papel crucial de criar oportunidades e buscar parcerias intersetoriais para desenvolver práticas emancipatórias em saúde ambiental^(4)^, a fim de resgatar um ideário de ética ambiental e de responsabilidade com os direitos à saúde^(7,9)^.

Em uma perspectiva sistêmica, plural e bioética, as competências do enfermeiro devem subsidiar a implementação de boas práticas de avaliação, monitoramento e redução dos riscos à saúde ambiental, tais como a implantação de processos de Enfermagem, diagnóstico participativo socioambiental e educação cidadã, considerando as questões do território e da saúde global^(4,7,10)^.

Faz-se necessário, então, que diferentes associações e instituições de educação e de saúde possam proporcionar atividades e eventos que estimulem o debate, a sensibilização e a percepção crítica de estudantes e profissionais da Enfermagem sobre temáticas relacionadas à saúde ambiental e ao desenvolvimento sustentável, assim como as proposições da ANHE^(14)^, ANHE Latinoamérica^(9)^ e da Associação Brasileira de Enfermagem em 2023^(35)^. As propostas de universidade sustentável e universidade promotora da saúde são, também, exemplos que vêm contribuindo para imbuir pensamentos e atitudes éticas de saúde ambiental no contexto universitário^(36,37)^.

Para tanto, urge a promoção de mais espaços nas universidades que forneçam subsídios efetivos para o percurso de uma formação emancipatória do enfermeiro como um agente crítico e estratégico de mudanças da realidade, com capacidade de inovar, desenvolver, adaptar e prospectar novos estudos, modelos, políticas e práticas de cuidado comprometidos com a saúde, a ética ambiental e a sustentabilidade da vida, perante os desafios emergentes impostos pelos impactos ambientais na saúde local e global.

### Limitações do estudo

Este estudo possui limitações. Destaca-se o fato de ter sido realizado com um único grupo de graduandos de Enfermagem de uma instituição de ensino superior. Outra limitação refere-se à utilização da abordagem metodológica qualitativa e aos resultados baseados nas percepções subjetivas dos estudantes, que foram desvinculadas de uma avaliação objetiva sobre o alcance das competências profissionais por eles informadas.

### Contribuições para a área da Enfermagem

O estudo contribui para salientar a necessidade de reformulação curricular, visando à integração mais efetiva dos conceitos e práticas cuidativas em saúde ambiental na formação de enfermeiros. Outrossim, destaca-se a importância de sensibilizar os graduandos de Enfermagem sobre questões contemporâneas e complexas de saúde ambiental, tais como a justiça ambiental e a equidade em saúde, a fim de fomentar práticas mais conscientes, inovadoras e ecoéticas dos futuros profissionais de saúde, com impacto nos cenários locais e globais.

Destarte, a pesquisa está alinhada ao compromisso de fortalecimento da formação e das práticas profissionais de Enfermagem em saúde ambiental e tem potencial para contribuir para o aprimoramento da abordagem interdisciplinar nos currículos, enaltecendo o valor da ciência, da educação e da profissão da Enfermagem no contexto da saúde global, da saúde pública e meio ambiente no Brasil e nas Américas.

## CONSIDERAÇÕES FINAIS

Mudanças curriculares, como a inclusão de práticas interdisciplinares de ensino em saúde ambiental, foram compreendidas pelos estudantes como indispensáveis à formação dos enfermeiros. As reflexões deste estudo acerca das potencialidades de se adotar estratégias curriculares em saúde ambiental nos contextos formativos do enfermeiro corroboram o caminho rumo ao rompimento da práxis educativa baseada em ações e metodologias meramente informacionais e instrumentais, e ao despertar para o olhar crítico e a atitude solidária de futuros enfermeiros na busca por sociedades mais justas, sustentáveis e saudáveis.

## Data Availability

Os dados da pesquisa estão disponíveis no corpo do artigo.
